# Late onset psychosis as an indicator of c9orf72 frontotemporal dementia: a case report

**DOI:** 10.1192/j.eurpsy.2025.1736

**Published:** 2025-08-26

**Authors:** M. Irureta, I. Loinaz, M. Alonso

**Affiliations:** 1 Hospital Universitario Donostia, Donostia-San Sebastián; 2 Universidad de Barcelona, Barcelona, Spain

## Abstract

**Introduction:**

Frontotemporal dementia (FTD) is a heterogeneous group of brain disorders associated with progressive frontal and temporal lobe atrophy. The main syndrome, behavioral variant FTD, is characterized by personality and behavior changes such as apathy, disinhibition, loss of empathy, new compulsive behaviors and executive dysfunction (Pressman, 2021). Psychotic symptoms may occur years prior to diagnosis of FTD, which can lead to misdiagnosis of a psychiatric disorder.

**Objectives:**

To highlight late psychosis as an initial indicator of frontotemporal dementia and emphasize the relationship of somatic delusions with the c9orf72 mutation.

**Methods:**

We reported the clinical case of a 64-year-old patient who was admitted in our acute psychiatric unit for late-onset psychosis with predominance of somatic delusions as the first indicator of c9orf72 frontotemporal dementia. Access to the medical history of the patient and non-systematic review of the literature on MEDLINE (Pubmed) was conducted.

**Results:**

A 64-year-old male initially consulted the mental health services for oral stereotyped movements and behavioral changes after dental surgery. After a few months, the patient was concerned because he reported that he had holes in his mouth from which liquid was leaking. He consults numerous dentists and undergoes several surgeries, without success. The belief becomes more intense and delirious, and he develops self-harming ideation that leads him to be urgently admitted to the acute psychiatric unit. During the admission, aggressive behavior of the patient towards the staff was observed. Treatment with antipsychotics, antidepressants and ECT was carried with little improvement. He was admitted to a Medium-Term Unit where the patient deteriorated rapidly becoming apathetic, uninhibited and mutistic. Finally, after several months, he was diagnosed with C9orf72 frontotemporal dementia. Psychotic symptoms may be the initial manifestation of frontotemporal dementia. There is a hypothesis that somatic delusions are caused by an altered body schema, correlated with a pattern of posterior, subcortical and cerebellar atrophy (Ducharme, 2011).

**Conclusions:**

Late-life psychosis should be investigated as a possible prodrome of a frontotemporal dementia. Physicians should be aware given the psychiatric-like presentation, unaltered imaging exam and delayed appearance of typical symptoms of FTD. An increased prevalence of somatic delusions in FTD patient with C9orf72 expansion has been reported (Downey, 2014).

**Disclosure of Interest:**

None Declared

